# Behaviour change intervention for smokeless tobacco cessation: its development, feasibility and fidelity testing in Pakistan and in the UK

**DOI:** 10.1186/s12889-016-3177-8

**Published:** 2016-06-10

**Authors:** Kamran Siddiqi, Omara Dogar, Rukhsana Rashid, Cath Jackson, Ian Kellar, Nancy O’Neill, Maryam Hassan, Furqan Ahmed, Muhammad Irfan, Heather Thomson, Javaid Khan

**Affiliations:** Department of Health Sciences, University of York, York, YO10 5DD UK; Bradford Teaching Hospitals NHS Foundation Trust, Bradford, UK; School of Psychology, University of Leeds, Leeds, LS2 9JT UK; Department of Medicine, The Aga Khan University, Karachi, Pakistan; Cameos Consultants, Islamabad, Pakistan; Department of Public Health, Leeds City Council, Leeds, LS7 3NB UK

**Keywords:** Tobacco, Smokeless, Chewing, South Asian, Behavioural support, Behaviour change, Fidelity, Adaptation, Cessation, Feasibility

## Abstract

**Background:**

People of South Asian-origin are responsible for more than three-quarters of all the smokeless tobacco (SLT) consumption worldwide; yet there is little evidence on the effect of SLT cessation interventions in this population. South Asians use highly addictive and hazardous SLT products that have a strong socio-cultural dimension. We designed a bespoke behaviour change intervention (BCI) to support South Asians in quitting SLT and then evaluated its feasibility in Pakistan and in the UK.

**Methods:**

We conducted two literature reviews to identify determinants of SLT use among South Asians and behaviour change techniques (BCTs) likely to modify these, respectively. Iterative consensus development workshops helped in selecting potent BCTs for BCI and designing activities and materials to deliver these. We piloted the BCI in 32 SLT users. All BCI sessions were audiotaped and analysed for adherence to intervention content and the quality of interaction (fidelity index). In-depth interviews with16 participants and five advisors assessed acceptability and feasibility of delivering the BCI, respectively. Quit success was assessed at 6 months by saliva/urine cotinine.

**Results:**

The BCI included 23 activities and an interactive pictorial resource that supported these. Activities included raising awareness of the harms of SLT use and benefits of quitting, boosting clients’ motivation and self-efficacy, and developing strategies to manage their triggers, withdrawal symptoms, and relapse should that occur. Betel quid and Guthka were the common forms of SLT used. Pakistani clients were more SLT dependent than those in the UK. Out of 32, four participants had undetectable cotinine at 6 months. Fidelity scores for each site varied between 11.2 and 42.6 for adherence to content – maximum score achievable 44; and between 1.4 and 14 for the quality of interaction - maximum score achievable was 14. Interviews with advisors highlighted the need for additional training on BCTs, integrating nicotine replacement and reducing duration of the pre-quit session. Clients were receptive to health messages but most reported SLT reduction rather than complete cessation.

**Conclusion:**

We developed a theory-based BCI that was also acceptable and feasible to deliver with moderate fidelity scores. It now needs to be evaluated in an effectiveness trial.

**Electronic supplementary material:**

The online version of this article (doi:10.1186/s12889-016-3177-8) contains supplementary material, which is available to authorized users.

## Background

Smokeless tobacco (SLT) is a product containing tobacco, placed in the mouth or nose but not burned at the time of use [1]. Consumed worldwide, a wide variety of unprocessed (sun-dried), processed or manufactured products are available, which are either chewed, sucked or applied to gums and teeth [2]. SLT contains nicotine and carcinogenic nitrosamines [3] and each year leads to a loss of more than 6 million Disability Adjusted Life Years (DALYS) and a quarter of a million deaths [4]. This is due to a higher risk of oral, pharyngeal, and oesophageal cancers, and fatal myocardial infarction compared to non-tobacco users [4]. Furthermore, SLT use leads to gum disease and tooth decay [5]. In women, its use is associated with reduced gestational age, low-birth weight and stillbirth [6, 7] and a low bone-mineral density [8].

More than 300 million adults in at least 115 countries consume SLT [4]. However, its use is particularly common in South and South-East Asian regions [9]. For example, a quarter of adults in Bangladesh and India use SLT [10]. In Pakistan, 16.3 % males and 2.4 % females consume SLT on a regular basis [11]. It is also popular among South Asian diaspora in the UK; being particularly common among Bangladeshi-origin women (19 %) and men (9 %) [12]. Being considered as part of their cultural heritage and family tradition, smokeless tobacco has social and cultural acceptance among South Asians [13]. The habit is taken up at a young age as most families equate it to confectionary [14]. Peer pressure, family acceptance, medicinal use (for dental pain) [15], easy access [16], low price, lack of regulation, and media promotion all contribute to its widespread use [17].

Among those using SLT, 33-63 % think of quitting the habit on their own, although the majority of such attempts are unsuccessful [18–22]. The key factors that play a significant role in quit initiation and decision making among SLT users are social support from close family and friends, media depictions and advice from doctors/dentists [17]. Other studies have reported peer pressure and isolation as being the main reasons for relapse among those SLT users who are trying to quit [17]. A Cochrane review, concluded that behavioural support might be effective in achieving abstinence from SLT [23]. The review was unsupportive for the use of bupropion and inconclusive for nicotine replacement therapy (NRT). Varenicline has also been shown to achieve abstinence in people using smokeless tobacco [24]. However, these trials were mainly conducted in the US and European populations who, compared to South Asians, use less addictive and less hazardous products (e.g. moist snuff). Therefore, it is not possible to infer the effectiveness of behavioural support among South Asians in smokeless tobacco cessation; a knowledge gap acknowledged by NICE [25]. Moreover, its consumption among South Asians has strong socio-cultural dimensions, which need to be considered in behavioural support interventions. The non-trial data from the UK ‘Bangladeshi Stop Tobacco Project’ supports the use of behavioural support for smokeless tobacco [26]. However, the project did not document adapting behavioural support to the socio-cultural context of the target population. The cessation advisors in the UK, offering behavioural support to smokeless tobacco users of South Asian-origin commonly modify their advice, which is adapted from smoking cessation interventions, according to what they perceive appropriate. However, there are variations in practice, no standard treatment protocol for a culturally adapted intervention and no evidence for its effectiveness. In addition, Bangladeshi men using smokeless tobacco have reported being marginalised by NHS services [27].

Behavioural support interventions for tobacco cessation in minority ethnic groups are more likely to be effective if these take account of their socio-cultural context and are adapted accordingly. There are some helpful frameworks to guide systematic cultural adaptation of health interventions for minority ethnic groups [28]. Consensus has also emerged on the key domains of behaviour change useful for implementing interventions [29]. Behavioural determinants have been mapped across various behaviour change techniques (BCTs) [29]. More recently, a taxonomy of these BCTs, used and found effective in health promotion, has been updated [29]. Using these theoretical constructs, we aimed to: (i) develop a bespoke behaviour change intervention (BCI) for South Asians for SLT cessation by adapting an existing intervention for smoking cessation, and then (ii) assess the feasibility of delivering this intervention; recruiting and retaining participating sites and clients, and collecting data to inform a future trial. We also explored acceptability of the content and delivery of the BCI as well as its perceived efficacy.

## Methods

The methods used in developing and assessing feasibility of a behaviour change intervention (BCI) for SLT cessation followed the Development and the Feasibility/piloting phases of the MRC Framework for developing and evaluating complex interventions [30], as follows.

### Developing the BCI

Our BCI consisted of a selection of theory-linked BCTs appropriate for use in South Asian population to help them quit SLT. The development of the BCI was carried out in three steps: i) identification and selection of the key determinants of SLT use among South Asians and relevant BCTs, ii) translating a list of matched BCTs into intervention activities and developing a culturally appropriate resource to facilitate the delivery of these activities, and iii) a post-hoc refinement of this resource.

In the first step, a literature review was undertaken to identify key theory-based determinants that influence early uptake and continued use of SLT in people of the South Asian origin. Using the most up-to-date taxonomies of BCTs, these key determinants were mapped on to the techniques found effective in behaviour change in general [31] and in smoking cessation [32], in particular. For building consensus on the most appropriate BCTs, a modified Nominal Group Technique, in which experts express their views independently before these are fed back to the group (usually by means of a facilitator) for discussion, was used [33]. Two expert panel meetings were convened, inviting academics and practitioners with expertise in behaviour change and tobacco cessation. In meeting one, experts gave scores on a four-point scale to all SLT determinants on ‘strength of association’ and ‘modifiability’, and indicated their agreement on binomial scale (Yes/No) to the linked BCTs based on its’ ‘efficacy’, ‘acceptability’ and ‘deliverability’. Using an algorithm, scores for ‘strength of association’ and ‘modifiability’ were combined for every SLT determinant. For example, a determinant with no ‘strength of association’ and no ‘modifiability’ was given a score of 1, while on the other hand, another determinant with high ‘strength of association’ and high ‘modifiability’ was given a score of 10. Similarly for BCTs, no agreement on ‘efficacy’, ‘acceptability’ and ‘deliverability’ received a score of 1, while agreement on all three received a score of 4. Subsequently, average expert scores were calculated for all determinants and BCTs. In meeting two, a full list of determinants and BCTs was presented with their average scores. Cut-offs to select the most useful determinants and BCTs, were established. All determinants scoring 7 or above and all BCTs scoring 3 or above were included. Those scoring below 6 for determinants and below 3 for BCTs were excluded. Determinants scoring between 6 and 7 were discussed and included if deemed appropriate by the panel. Following consensus on what was to be included in BCI, the expert panel proposed how to translate the final list of determinants and BCTs into the intervention.

In the second step, key intervention activities were operationalised as a resource to be used by the tobacco cessation advisors. This phase of development took place in a small working group drawn from the expert panel and supplemented by experts in designing health promotion materials for minority ethnic communities. Using examples drawn from their experience of working with South Asian communities, the experts suggested how best to frame the intervention activities in culturally appropriate form and content. Mindful of the standard treatment programme for smoking cessation [34] and a desire to maintain standardisation, the BCI activities were categorised and the resource was structured into pre-quit, quit and post-quit sessions.

The final step in the development of the BCI took place after assessing its feasibility (see below) and consisted of a review of the activities and the form and content of the resource by the expert panel. In two expert panel meetings, participants drew on the field experience of delivering the BCI to refine the intervention.

### Feasibility of delivering the intervention

We assessed the fidelity to the BCI using a pre-defined index. This consisted of two sub-indices: the Adherence Index, for assessing compliance to the activities within the BCI and the Quality Index, for assessing competence with which the BCI was delivered. Both indices consisted of a number of items for scoring against a three-point Likert scale (0 = not implemented, 1 = partially implemented and 2 = fully implemented).

Fidelity data were collected for all participating clients at the pre-quit, quit and post-quit sessions by audio-recording their interactions with the advisors. This method of observation has the advantage of generating data that are potentially more objective than self-reports [35] and yet less obtrusive than direct (in-person) observations [36], which are likely to be susceptible to observation bias. Due to language variations in client-advisor interactions between different study sites, three separate bilingual (English and another South Asian language) coders were trained on scoring the fidelity index [37] by listening to audio-recordings. Participant engagement was assessed by estimating completion rates of the calendars offered at the quit session.

Fidelity data was summarised descriptively but no comparative statistical analysis was conducted. Participants receiving the BCI and completing the calendars were reported as counts and percentages. Fidelity scores and the duration of pre-quit, quit and post-quit sessions were presented as means and standard deviations by study sites (i.e. London, Bradford, Leeds, and Leicester in the UK, and, Karachi in Pakistan).

### Feasibility of recruiting and retaining participants

The feasibility study was conducted in four NHS Stop Smoking Services (Leicester, Leeds, Bradford and Tower Hamlets) in the UK and within a community setting in Karachi, Pakistan. Seven cessation advisors (4 in Tower Hamlets, 1 in Leicester, 1 in Bradford, 1 in Leeds) in the UK and one in Pakistan (based at Aga Khan University) participated in the study. All cessation advisors received a full-day training session on using the BCI.

A total of 32 adult (≥18 years) regular smokeless tobacco users (regular use = at least once in 7 days for 6 months or more) of south Asian-origin were recruited (16 in the UK and 16 in Pakistan). We excluded those who also smoked cigarettes and/or had attended cessation services in the past.

In the UK, the cessation advisors sifted through their referrals to identify potentially eligible clients and posted a participant information leaflet (translated in relevant south Asian languages – Urdu, Bengali, Hindi and Gujarati) to them in advance of their first (pre-quit) appointment. In Pakistan, potential participants were recruited through a voluntary sector organisation based in a community with high use of SLT. Outreach community volunteers identified potentially eligible clients and provided them with the study information. In both settings, informed written consent was taken at their first (pre-quit) appointment by our research team.

All participating clients who received the BCI completed a questionnaire at the pre-quit session (baseline) and provided a saliva sample for cotinine testing. Cotinine testing was repeated at 6 months with 28 of 32 participants completing this. The questionnaire included demographic information (e.g. age, gender, ethnicity, occupation, medical history), past and present smokeless tobacco use, past smoking history, family history, motivation to quit, and nicotine dependency using translated Oklahoma Scale for Smokeless Tobacco Dependence (OSSTD) [38], and Fagerstörm Tobacco and Nicotine Dependency scale for Smokeless Tobacco (FTND-ST) [39].

Sixteen client participants were also invited to take part in three semi-structured interviews after their pre-quit, quit and post-quit sessions. Participants were purposefully selected to ensure equal gender representation as well as participants from all five sites. They were given a £10 shopping voucher in the UK and a (equivalent value) food basket in Pakistan for their time. The advisors who had delivered the BCI were interviewed at the end of the study.

The aim of the client interviews was to get a detailed insight on: a) the acceptability of the content and delivery of the BCI, b) its perceived impact, and c) to make recommendations for improvement to the BCI. The aim of the advisor interviews was to explore a) the feasibility (barriers and facilitators) of delivering the BCI and b) make recommendations for the post-hoc refinement of the BCI. Topic guides were developed to mirror the delivery of the BCI during cessation sessions (Additional file 1). Interviews were conducted at GP surgeries, participant homes and community centres in the UK, and at a community outreach centre in Pakistan.

The interviews were audio recorded, translated (Pakistan interviews) and transcribed verbatim. The interview data were analysed using content analysis [40]. A categorisation matrix was developed, based on the aims of this qualitative component and the structure of the BCI, to which all of the client and advisor data were coded by a team of three researchers (two UK and one in Pakistan). The data within each category (e.g. Products Talk, Triggers Talk) were then reviewed and discussed by these researchers before being written up. Data for the client and advisor participants were reviewed separately and then brought together at the write up stage. Recommendations for the post-hoc refinement of the BCI were then taken to the final expert panel workshops for consideration.

### Feasibility of collecting data to inform future trial

Baseline data was summarised descriptively by study country (i.e. the UK and Pakistan) but no formal statistical comparisons were undertaken. Continuous measures (e.g. dependency scores) were reported as means and standard deviations while categorical data were reported as counts and percentages. Based on the saliva cotinine levels, a cut-off of 15 ng/ml [41, 42] was taken as confirmation of abstinence from smokeless tobacco use. The 6-month saliva cotinine results were dichotomised using the cut-off and results of quitters versus continuing users, and were summarised taking in account the baseline levels of saliva cotinine.

## Results

### Developing the BCI

A recently published systematic review provided us with a list of key determinants of SLT use among South Asians. Those selected by the expert panel were, lack of awareness about tobacco and other ingredients contained in the SLT product, lack of information on the health effects of SLT use, lack of knowledge and misconceptions on the outcomes expectancies, lack of knowledge and ability to self-control (have a self-image, lack of incentives to quit, lack of self-efficacy to quit and lack of skills to resist it), and socio-cultural norms (role models using it and easy access at home and shops). Other determinants relating to public health issues such as media influences, lack of regulation and cessation support, low cost of the products, and socio-cultural norms such as peer pressure and fear of isolation were deemed outside the scope of individual face-to-face behavioural intervention and thus, were excluded. The only BCTs excluded were the material incentives/rewards for ‘lack of incentives to quit’ and restructuring the physical environment for ‘easy access at home and shops’; further details on the BCTs included can be found in the BCI resource (called BISCA resource).

The BCI resource consisted of an advisor pack (practice manual and interactive cards) and a client pack (take-home booklet and 4-week calendar), organised into pre-quit, quit and post-quit sessions. The advisor pack provided a set of different scenarios using photographic materials inviting a dialogue between the advisor and the client. This utilised relevant BCTs focusing on: knowledge about tobacco contained in the products, consequences of using it and stopping its use, myths busting; assessment of quit history and readiness to quit; self-regulatory capacity/skills to deal with tobacco dependence by facilitating, strategies to manage the triggers and withdrawal symptoms, monitoring progress and setting self-incentives. The take-home booklet was a copy of the interactive cards meant for reinforcement of the messages delivered in the session, while the calendar was (offered at the quit session) designed for self-monitoring.

After piloting the BCI, relatively minor changes were made. The changes in its content included modifications in selected images for cultural appropriation, additional slides on preparation and planning for the quit date and management of withdrawal symptoms. The structure of the interactive cards was changed to a flipbook format. Information relating to quit attempt, which was prompted by the cards, was integrated within a client record form used by the services. In the flipbook, the key messages and brief instructions for delivering each image slide appeared on its back facing the advisor for practical purpose. The final modifications in the BCI resource were presented to the expert panel in meeting four, and consensus reached. It was also agreed to develop a digital application of the BCI resource for broader outreach.

### Feasibility of delivering the intervention

The fidelity index consisted of 29 items - 22 in the Adherence Index, representing BCI content and seven in the Quality Index, representing competence with which the BCI was delivered.

**Fig. 1:**
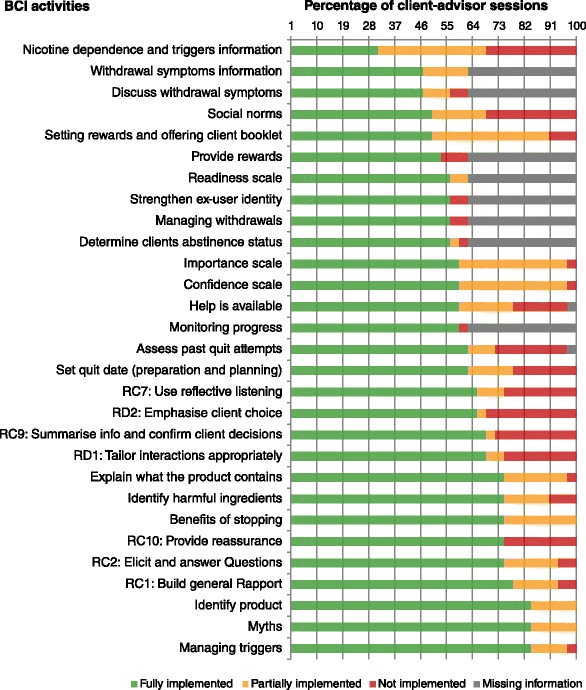
Delivery vs. Intention - the proportions of client-advisor sessions in which different intervention components were delivered

The intervention activities related to identifying SLT products, busting myths and managing triggers were fully implemented in more than 80 % of the client-advisor sessions, followed by explaining what the product contains, identifying harmful ingredients and management of triggers - fully implemented in about 70 % of these sessions (Fig. 1). The intervention activities related to the information on nicotine dependence, triggers and withdrawals, setting rewards, offering client booklets and the scales on importance, confidence and readiness were the least implemented ones (either not implemented or only partially implemented); for further detail refer to Additional file 2. Twelve out of 16 participants from Pakistan completed and returned the self-monitoring calendars, however, these were only offered to three participants in the UK among whom none were collected at the follow-up.

Across the five study sites, the scores for the Adherence Index ranged from 11.2 (SD 2.1) to 42.6 (SD 1.9) - maximum score = 44; while the scores for the Quality Index ranged from 1.4 (SD 0.9) to 14 (SD 0) - maximum score = 14, (Table 1). Similarly, the duration of pre-quit sessions varied from 4 to 54 minutes, quit sessions from 5 to 35 min and post-quit sessions from 2 to 22 min, between the five study sites.

**Table 1:** Average fidelity index scores and duration of BCI sessions, by study sites

Site ID	Fidelity index scores			Duration of sessions in minutes					
	Mean (SD^a^)			Mean (SD)					
	N^b^	Adherence Index	Quality Index	N	Pre-quit	N	Quit	N	Post-quit
1	5	23.8 (8.4)	11.6 (5.4)	5	23.3 (19.1)	1	12.0 (.)	2	11.4 (2.8)
2	5	11.2 (2.1)	1.4 (0.9)	5	24.1 (4.1)	0	.	0	.
3	2	29.0 (16.9)	10.0 (5.7)	2	41.4 (18.4)	1	35.2 (.)	1	21.4 (.)
4	4	14.8 (3.2)	6.8 (3.8)	4	22.0 (4.7)	1	5.6 (.)	2	7.9 (5.1)
5	16	42.6 (1.9)	14 (0)	16	30.8 (7.6)	16	8.7 (2.9)	15	3.6 (1.5)

^a^SD is the standard deviation of the mean

^b^N is the number of participants with data available

### Acceptability of the BCI and its Perceived Impact

#### Content

On the whole, the content of the intervention was perceived positively by clients and advisors in both countries. The majority agreed that the use of images on the interactive cards were helpful in portraying behavioural change messages. Clients demonstrated a particular interest in the harmful ingredients card and associated discussion. They commented that this had increased their knowledge about the harms of SLT use which many in their community may not be aware of due to the prevailing myths and misconceptions about SLT products (Quote BRA004/a, Table 2).

**Table 2:** Illustrative Quotes

ID	Quote
BRA004/a	A: I, I wouldn’t say I actually believe them, but my mother-in-law did say if you eat paan with tobacco it strengthens your teeth, cos she, her teeth are fine, she got no cavities, they’ve stained a lot, but my mum; when I was small I used to have travel sickness so she used to say, you know, just the beetle nut on its own, cos it dries out your mouth, cos when you feel sick, you know that tangy, liquidy thing you get in your mouth?
	Q: Yeah.
	A: So when you take that it dries out your mouth so you don’t be sick; and I did used to take it, I can remember, yeah, so.
	Q: Yeah. Is that a common belief amongst Bengali communities?
	A: Yeah, it is. But they said, you know, if you have, my mother-in-law says if you have, you know, tooth aches or pains in your tooth or gum to chew with tobacco. She’s told me, she, cos I’m, I have very weak gums and she tells me “Oh, you know, the raw tobacco leaf” she says “oh eat with that”. I said “No mum, I can’t eat that cos it’s really, really strong”. Cos with this, it’s not just tobacco, there’s a lot of other things in it like that, so.
	Female Client, UK
APK001/a	Like, yeah, to grade their readiness. And, OK, so, OK, and with card number one, I don’t think this really represented the type of tobacco they used very well; cos I like, I showed them this card and I was like “Which one do you use?” And they would say “Oh we use gutka, we use naswar”. And then I’d point to gutka in the picture, I’d point to naswar, I’d say “Oh this is naswar and this is gutka”. And they’re like “No, but this isn’t the type we use” and everything. So maybe I, I think maybe this was more oriented towards the England wing of the study where like this is what it looks like over there, whereas over here it doesn’t really look like that… it’s not very all, it’s not all that commercial, like everyone makes their own at home and everything, so it’s not very packaged, very professionally.
	Advisor, Pakistan
BRA004/a	Yeah, this is, just telling you what’s in it, yeah, that I think they will really take notice of that card, and you know like if you have actual pictures of actual things that can happen; cos in one of the tins that come from Bangladesh recently it’s got the picture, her grandfather gave to her father, it’s literally a man with a picture of cancer in his mouth with a big tumour. Every time I pick it up I just put it down; and she says “Chuck it away, chuck it away”. Cos you know if, if you have pictures like that it literally does make you not want to take that.
	Female Client, UK
APK001/b	Yes, that was important to a certain extent, where I’d point out that “Oh, you know, once you stop smoking your teeth will look better, you will feel better”. And that one was a factor in certain patients where they, because we’re a population that’s obsessed with the tone of our skin, so they were just obsessed that, oh yeah, we will, like when I wasn’t using tobacco I used to be fairer so I’m gonna become fairer now and my teeth are gonna look better and everything; and then when some of them did stop and they came back for their third session their teeth did look better, they were very proud, proud of that fact, oh look at our teeth, they look so nice and everything. And then there was actually one client that even went and got his cleaned out and everything afterwards; so that was, yes.
	Advisor, Pakistan
APK001/c	Yes and no, because I feel like the main driving force behind the fact that most of the patients were motivated to quit was because we were motivating them. Like most of the patients were motivated because we talked about, oh OK, so tell me why you wanna quit? So most of them wouldn’t bring up the fact that it causes mouth cancer, they’d be like, oh we, because we’re spending too much money on this and we’re poor and we wanna pay our children’s school fees and everything. So that was why this was so effective, because they weren’t leaving it because they were scared, because I feel like that’s scare factor, and like maybe even as a doctor, like you can only scare patients so much until that becomes redundant and just becomes, OK fine, so maybe this just might happen, so what, you know?
	Advisor, Pakistan
LEI002/a	For me, I guess you, you know, you’ve got the health one there, that, that’s probably the main important, maybe emphasise a bit more on that and, and what, what are the harmful effects. For example, one of the things that made me go to the dentist is because my teeth discoloured and my gums started to drop as well. So after a lot of use, he’s told me that chances are the gums won’t grow again, but he’s certainly got’em all clean, and thank goodness, I’m grateful that I didn’t have any illness, cos I thought I had an illness on the back of that.
	Male Client, UK
ATH002/a	This [card10] is a good one, I think, yeah, sometimes when you talk to them you can’t always explain it, but these, these pictures are quite self-explanatory, like people can look at it and understand what other things they can do to, that will help them give up here… These, these are very good ones, these are very good ones. So maybe you can, for women you can think of like, you know, for Muslim or reading Koran or, or like if they’re a bit younger then maybe going to swimming; because they do love swimming, a lot of people love going to swimming; so maybe we can put that.
	Advisor, UK
APK001/d	Maybe they needed a better idea of what a reward meant, like I felt like I had to pump them a little; like “OK, a present for yourself, like you can get new clothes.” “Oh yeah, we’ll get some.” But then they were just like agreeing with whatever I was saying, and then when I asked them later on they didn’t, they didn’t even understand what I, what I was trying to ask them.
	Advisor, Pakistan
ALEE001/a	For some people it will be. I think maybe for a lot of Bangladeshi women it’s, not necessarily, cos I, I think they find it hard to know what they would reward themselves with, so, cos they wouldn’t actually go out and buy loads of clothes or; we did talk about maybe having a massage or something like that, I think. I think I suggested possibly because of the, she’s not gonna have the lip staining, maybe buying a lipstick (laughs) or something. But I, I think she found it really difficult to think of a reward; so we didn’t really discuss that, I don’t think.
	Advisor, UK
ALEI001/a	I think just to give it, them just a couple of weeks; abrupt is usually better in a, in some ways, because there’ll never be a right time or a date for them to stop, but I think just to give them just those two weeks, just to, for them to think about it and absorb everything what we’ve talked about, I think that will give you a result instead of just saying this is it and people don’t really relate to that
	Advisor, UK
ATH002/b	Only behaviour support might not work because none of us are trained counsellors; even the trained counsellors would find it difficult, with the counselling, to change somebody’s behaviour, and within two, four weeks or eight weeks would not be possible. So I think you do need the, the NRT products, they, they play an important role and a big part, and people think oh it’s like medicine, that might help me; not, it’s not true for everyone but for eighty percent, eighty to ninety percent of our client they go for the NRT support and it does help them and, or sometimes it could be psychological, you know, they think that, OK.
	Advisor, UK
ATH001/a	I think a lot of clients they tried to give up through will-power and they haven’t succeeded. Obviously because of my previous experience, NRT has been effective and quite a lot of clients they want some form of product to help them stop chewing tobacco because otherwise how the perception is, “if it’s just behavioural support I can just get my friend to support me, I don’t need an advisor”, so I think they just want a combination of maybe NRT and behavioural support.
	Advisor, UK
ALEI001/b	I think the cards itself are too simple and sometimes you can probably do it in verbal with the client and you don’t really need to show them a picture, I feel, but sometimes it does jog a memory; some things they would say that I’ve, I’ve experienced, they would say I probably haven’t even noticed…. Optional I would say; if it works for some people, fine, but I think you, it shouldn’t be a necessary that you need to use them, yeah.
	Advisor, UK
ATH001/b	Um I would say maybe a bit of both but I think a lot of clients they’re short for time, so they’re not able to spend that much time with you and, yeah I think the cut down definitely on the resources, make it shorter and maybe, I don’t know, maybe group things together in a nutshell.
	Advisor, UK
ALEI001/c	I think it was just off-putting using the, the, this resource with the card, for me personally, I think it was too confusing. I think if you got the assessment form done it would be much easier…. For me it isn’t working, it was too complicated. I couldn’t give, I felt I couldn’t give the, the client all that attention that I would normally give. As a smoker, let’s say, we would look at them and while they’re talking we’ll be going through the form and remembering what they’ve said so you’re not duplicating questions as well, so you’re just writing, everything’s on the form, any additional you can write, you know, down. So, you know, it’s like a memory isn’t it then? Maybe we can have the handbook just for ourselves and, but assessment form is really essential.
	Advisor, UK
APK001/e	So I think that way the resource was great. The only issue that I think the participants had and I think with, and maybe I has as well, with the resources, when we have to ask them to grade, like for example, like oh tell me from 0 to 10 how ready do you think you are to stop smoking this and everything; so the thing was they weren’t quite understanding this grade skill, and I think that’s just an issue with this resource; I think even as a doctor, when we ask patients about like to grade their pain on a pain scale, they face the same issue where they, they automatically just pick 10 or they pick 0, there’s no middle ground in the middle, yes… So I had this issue with one of the participants, the one, he didn’t actually commit to quit, so, all right, he didn’t actually quit, so, but then I was like “Oh how ready are you to stop using tobacco and everything?” And he said “10, 10, 10”. And then at the end of it I was like “OK, so are you ready to stop now?” And he was like “Oh no, I’m not ready to stop”. (Q laughs) …maybe if we had like a reference for each number maybe he would have understood more, maybe he would have told me, oh 4 or 3, 5 or something like that, so yeah.
	Advisor, Pakistan
LEI002/b	As I say, it’s a first time that I’ve come into something like this; so it’s been helpful that I can actually admit to the fact that I am addicted and I need to do something about it and, as I said, I’m, I’m reliant on, I’m gonna rely on the things that she’s offering me, that it’ll help me in my journey.
	Male, Client, UK
APK001/f	Yeah the majority of them had quit for like a few days, right, so when they came at this point in time they were experiencing very, very severe withdrawal symptoms, like I mean I’ve seen people in withdrawal but those are very similar. So there is this one theory that I have, and like it’s, this story is based on the information I’ve got from the patients, they say that the tobacco they have, or whatever form they have it in, it’s just not tobacco, there are several other illicit substances involved. So one of the men actually claimed that there was, what he says was (genus?) in it, which I, I think that’s heroin or cocaine in it or something like that, but this is just one person saying this to me. So that was an issue at this point because some of the women who quit, they were just visibly dizzy, like they were just sitting there and they were like this, like I could tell (Q laughs) that they were out of it really, so yeah, like one was just like just not even there, like she was barely comprehending what I was saying, you know, in the end like she actually, we had to actually take a break in the middle of it. “OK, how about you take five and everything, go and take a glass of water.” But they were visibly, visibly, visibly out of it; so they were experiencing very severe withdrawals… Maybe this can be like a potential like confounding factor where we’re gonna have a lot people coming here and who don’t quit. Because if there are other illicit substances involved, because our resource is only for tobacco, you know, and like when it comes to like whatever illicit substance is there like, you know, even if it’s like cocaine, heroin, marijuana like you need other resources there, you know, like maybe you have to give them medication or like they need to go to hospital or something like, but by the end of it, four weeks later it was not a factor I think, like, yeah.
	Advisor, Pakistan

Some clients in Pakistan were unable to relate to a few images. For example, the images showing commercially packaged SLT products, which are not sold as such in Pakistan. (Quote APK001/a, Table 2).

Similar to current evidence [43], some advisors and clients in the UK felt that there was a need for more images of negative health consequences alongside positive images of quitting. For example, images of oral cancer or dental problems similar to those used on cigarette packaging might increase clients’ motivation to quit (Quote BRA004/a, Table 2). In contrast, clients in Pakistan felt that there should be more emphasis on an improved appearance after quitting SLT use (Quote APK001/b, Table 2).

For clients in Pakistan, the main benefit of quitting SLT use was seen to be financial (Quote APK001/c, Table 2), whereas in the UK, the emphasis was placed on health benefits (Quote LEI002/a, Table 2).

Most clients could relate to the examples for managing urges and avoiding triggers. They found the information and the coping strategies helpful and useable (Quote ATH002/a, Table 2). On the other hand, the concept of rewarding oneself after quitting had divided opinions. Although the picture card on rewards were seen positively; the majority of clients did not set a reward for themselves following the quit attempt. The advisor in Pakistan suggested that the majority of clients did not understand the concept of rewarding oneself (Quote APK001/d, Table 2). Similarly, in the UK one client suggested that rewarding oneself in this way is not a concept that the community can identify with (Quote ALEE001/a, Table 2). Improved health was seen as a reward in itself.

#### Delivery

Intervention delivery varied as UK advisors incorporated the BCI into their current modes of practice whereas in Pakistan, as there was no pre-existing cessation service, the intervention was delivered as a standalone activity. In the absence of any specific guidelines for SLT cessation, most UK advisors felt that a gradual reduction worked better than an abrupt cessation (which was the approach advocated in the BCI) (Quote ALEI001/a, Table 2)). All the UK advisors and the majority of the UK clients felt that information on nicotine replacement therapies (NRTs) should be included in the BCI as it is routinely offered to clients (although there is no scientific evidence of their effectiveness for SLT users) [43] and is an important part of the service that they offer (Quote ATH002/b, Table 2). Advisors felt that prescribing or advising on NRTs further motivates clients to quit (Quote ATH001/a, Table 2). Participants in Pakistan did not comment on this as NRTs are not currently available there.

Some UK advisors argued that the use of picture cards should only be used if the advisor deemed it necessary. They felt that for some clients, the resource might be delivered verbally only (Quote ALEI001/b, Table 2).

Finally, many UK advisors suggested changing the structure of the resource to improve its deliverability and reduce the duration of the pre-quit session, which some clients also felt, was too long (Quote ATH001/b, Table 2). A few also suggested that combining the handbook and the interactive cards into a single tool might facilitate intervention delivery. A few of the advisors in the UK stressed that there is a need for an assessment form to complement the resource (Quote ALEI001/c, Table 2).

#### Perceived efficacy

All clients reported that they felt highly confident and motivated to quit SLT scoring seven and above (scale of 1–10) on a confidence and readiness to quit scale at the pre-quit and quit sessions. However, there were some concerns among advisors regarding the scales who felt that clients didn’t necessarily understand what they represented and were simply scoring highly to please the advisor (Quote APK001/e, Table 2).

There was also strong motivation among clients following the pre-quit session to quit SLT use based on the information they received in the session (Quote LEI002/b, Table 2). After the post-quit sessions, the majority still emphasised a general desire and ability to quit however some described the challenges of quitting abruptly and had relapsed or decided on a gradual reduction approach rather than abstinence. Clients in Pakistan particularly reported a high degree of withdrawal symptoms, which may have impacted on their motivation to remain abstinent (Quote APK001/f, Table 2).

#### Recommendations for improving the BCI

Client and Advisor suggestions for refining the BCI are presented in Table 3 along with post-hoc refinement that occurred.

**Table 3:** Recommendations for refinement of the BCI

Element	Recommendation	Implementation
Layout and structure	Advisors in the UK stressed that there is a need for an assessment form to complement the resource (ALEI001/d).	We have developed a form where advisors can record information about their clients to complement the resource and assist with data collection for their services.
Layout and structure	Advisor participants suggested combining the handbook and picture cards together to improve deliverability.	We restructured the resource and developed a flipbook so that the guidance note for the advisor appears at the back of each picture card.
Content	The majority of UK participants felt that information on nicotine replacement therapies (NRTs) should be included in the resource as it is routinely offered to clients and is an important part of the service that they offer (ATH002/b). Advisors felt that prescribing or advising on NRTs further motivates clients to quit (ATH001/b). Participants in Pakistan did not comment on this as NRTs are not currently available there.	AS there is no scientific evidence of the effectiveness of NRTs in SLT users, we have not included NRT support at this stage within the resource.

A number of other suggestions were made on the contents from participants throughout the BCI. All these detail specific recommendations were incorporated within the BCI when refined

### Feasibility of recruiting and retaining participants

We successfully recruited all 32 participants, 16 in the UK (5 in Tower Hamlets, 5 in Bradford, 2 in Leeds, 4 Leicester) and 16 in Karachi, Pakistan. All 32 participants received the intervention and provided baseline data including saliva sample for cotinine test. However, only 64 % (23/36) completed full six-month follow up - 10/16 in the UK and 13/16 in Pakistan.

### Feasibility of collecting data to inform future trial

Baseline questionnaires took 30–60 min to complete. All questionnaires in Pakistan were completed in full. In the UK, 12 questionnaires were completed in full and 4 were partially completed due to participant time constraints. There were some data missing from several sections; for the Fagerström Scale, four UK participants did not complete any of the scale and one participant missed one item on the scale for which imputation was carried out by averaging the responses to the same item by all participants. The score (within Fagerström scale) was imputed for this item before summarising the overall dependence score. 9.4 % (*n* = 3) were missing for aids to help quit and 3.1 % (*n* = 1) for intention to quit, 18.8 % (*n* = 6) for advice to stop using SLT and assistance to stop. We were able to collect the fidelity data from the audio recordings for 32 participants at the pre-quit, 19 at the quit and 20 at the post-quit sessions.

Table 4 compares the characteristics of the study participants at the two study sites. Nearly all participants in the study used SLT on a daily basis (*n* = 30 [93.8 %]). In Pakistan, a higher quantity of SLT used per day (mean 17.7 [SD: 12.34]) compared to the UK (mean 9.56 (SD: 9.65)). The two most popular forms of SLT were tobacco leaf or tobacco leaf mixture in the UK, and Gutka or tobacco, betel nut and catechu mixture in Pakistan (Table 4).

**Table 4:** Descriptive statistics of participant characteristics

Characteristics	Total	UK	Pakistan
Total, N	32	16	16
Sex, n (%)			
Male	16 (50)	8 (50)	8 (50)
Female	16 (50)	8 (50)	8 (50)
Current SLT use daily vs. non-daily, n (%)			
Daily	30 (93.8)	15 (93.8)	15 (93.8)
Non- daily	2 (6.3)	1 (6.3)	1 (6.3)
Current SLT use times per day, mean (SD)	13.6 (11.7)	9.56 (9.7)	17.7 (12.3)
Type of SLT used, n (%)			
Tobacco leaf or tobacco leaf mixture	11 (34.4)	8 (50)	3 (18.8)
Gutkha or tobacco, betel-nut & catechu mixture	23 (71.9)	7 (43.8)	16 (100)
Urge to use SLT, n (%)			
Not at all	4 (12.5)	0 (0)	4 (25)
A little of the time	5 (15.6)	1 (6.3)	4 (25)
Some of the time	14 (43.8)	7 (43.8)	7 (43.8)
A lot of the time	6 (18.8)	5 (31.3)	1 (6.3)
Almost all the time	2 (6.3)	2 (12.5)	0 (0)
All the time	1 (3.1)	1 (6.3)	0 (0)
Strength of urges for SLT, n (%)			
No urges	4 (12.5)	0 (0)	4 (25)
Slight	1 (3.1)	1 (6.3)	0 (0)
Moderate	10 (31.3)	6 (37.5)	4 (25)
Strong	12 (37.5)	6 (37.5)	6 (37.5)
Very strong	5 (15.6)	3 (18.8)	2 (12.5)
Extremely Strong	0 (0)	0 (0)	0 (0)
Fagerström Scale - SLT, n (%)			
Very Low (0–2)	2 (6.3)	2 (12.5)	0 (0)
Low (3–4)	5 (15.6)	5 (31.3)	0 (0)
Moderate (5)	3 (9.4)	2 (12.5)	1 (6.3)
High (6–7)	8 (25)	2 (12.5)	6 (37.5)
Very high (8–10)	10 (31.3)	1 (6.3)	9 (56.3)
Quit intentions, n (%)			
May quit in future, but not in 6 months	9 (28.1)	0 (0)	9 (56.3)
Will quit in the next 6 months	10 (31.3)	4 (25)	6 (37.5)
Will quit in the next 30 days	12 (37.5)	11 (68.8)	1 (6.3)
Quit attempt in last 12 months, n (%)	11 (34.4)	8 (50)	3 (18.8)
Use of any tobacco cessation aids, n (%)	2 (6.3)	2 (12.5)	0 (0)
Doctors asked about SLT use, n (%)	13 (40.6)	3 (18.8)	10 (62.5)
Doctors advised to stop SLT use, n (%)	10 (31.3)	3 (18.8)	7 (43.8)
Doctors offered aids to quit, n (%)	1 (3.1)	1 (6.3)	0 (0)
Smoked 100 cigarettes in lifetime, n (%)	1 (3.1)	1 (6.3)	0 (0)
UK population – saliva cotinine concentration ng/ml		Baseline	Follow-up
N		16	10
Min		1.0	0.1
Max		577.1	609.0
Mean (SD)		237.1 (194.62)	174.3 (220.7)
Pakistan – urine cotinine level (cotinine equivalent ng/mL)		Baseline	Follow-up
0 (1–10)		0	0
1 (10–30)		0	0
2 (30–100)		1	0
3 (100–200)		0	0
4 (200–500)		0	6
5 (500–2000)		0	1
6 > 2000		15	6
Missing		0	3

Participants in Pakistan appeared to be more addicted to SLT, with 56.3 % (*n* = 9) scoring very high and 37.5 % (*n* = 6) scoring high on the Fagerstrom dependence scale, compared to the UK, where 6.3 % (*n* = 1) participants scored very high and 12.5 % (*n* = 2) scored high (Table 4). For all participants where some answers were not given on the scale, imputations were performed.

All participants intended to quit, although these were over different time frames (Table 4). 34.4 % (*n* = 11) have attempted quitting in the last year (UK *n* = 8 [50 %], Pakistan *n* = 3 [18.8 %]). Some participants, especially in Pakistan, were being asked about their SLT use and encouraged to stop (Table 4), however, only one participant (3.1 %) was offered any support to stop using SLT, this participant was from the UK.

Cotinine measurement was done at baseline and at follow-up at 6 months with one participant in Pakistan and six in the UK not completing the follow up. Cotinine levels were found to be higher in the Pakistan population. In the UK, the mean saliva cotinine was 237.1(SD: 194.62) ng/mL. In Pakistan, urine cotinine sticks were used, with all but one participant having a level 6, the equivalent of >2000 ng/mL. At the 6 month follow up only 10 participants in the UK took the 6 month follow up test, with a lower average mean score of 174.3 (SD: 220.7). In Pakistan, 13 participants completed the follow up. There were no delays in data collection concerning baseline or follow-up, including the cotinine testing.

In Pakistan, all of the planned interviews (*n* = 8) were conducted following the pre-quit, quit and post-quit sessions. In the UK all of the planned interviews (*n* = 8) were conducted at pre- and post-quit. However, following the quit sessions, only two interviews were completed. This was because most of the UK advisors did not set a quit date with their clients and preference was given to a gradual reduction in SLT use rather than an abrupt cessation. As such six participants did not receive quit sessions. Eight advisors were interviewed (seven in UK and one in Pakistan) who had delivered the BCI at least once with clients.

## Discussion

Our study is the first attempt to design and conduct an early-phase evaluation of a bespoke behaviour change intervention for SLT cessation among people of South Asian-origin. Literature searching and expert consensus helped us to identify the BCTs required to make a tobacco cessation intervention more effective, feasible, and acceptable for South Asians. Design experts and tobacco cessation advisors working with South Asian communities assisted us in translating these techniques into an interactive and culturally appropriate educational resource, both in paper and electronic format. When we implemented this intervention albeit in a limited number of clients, we found high levels of fidelity in both adherence to the specified BCTs and quality-related criteria and most BCTs were implemented to their full extent. Both advisors and clients found the content of the intervention appropriate and mode of its delivery feasible. Most clients found images in the resource materials motivational and messages helpful in making an intention to quit. The intervention also appeared to boost their confidence in their abilities to quit and provide them with strategies to cope with their nicotine urges. Our study also showed high levels of clients’ engagement with the intervention, their ability to relate to resource images and their willingness to participate in related research activities. Differences were also observed between clients in Pakistan and in the UK particularly in relation to levels of nicotine dependence, intention to quit, previous quit attempts and cessation advice received in the past. While mean cotinine levels among our participants show a slight reduction at the follow-up compared to baseline, a higher maximum range indicates that a few might have increased their SLT use (Table 4). However, given the sample size insufficiency, no statistical inference should be made based on these findings.

While we found many similarities between our findings and those from studies of cigarette smoking, we also observed interesting differences between the two. A comprehensive evidence review of BCTs highlighted that identifying triggers for smoking and strategies to cope with these triggers are effective techniques for smoking cessation [44]. Likewise, our clients also found that acknowledging potential triggers for using SLT and strategies to deal with these triggers were helpful in building their self-efficacy for quitting. Concerns about stigmatising of certain social classes by depicting a negative portrayal of a smoker [45] has led to an increased emphasis on a positive image of an ex-smoker [46] in anti-smoking educational materials. However, our client cohort found negative portrayal of SLT users and graphic images of the health effects of SLT use compelling and motivating. This is also inline with the evidence that suggest that emotive and graphic imagery in anti-smoking campaigns is effective in generating an emotive response and an intention to quit [47–49]. Our findings highlight that a negative portrayal of SLT use could also be useful in ‘myth busting’ and challenging beliefs, often rooted in culture and lived experience, about the ‘positive’ expectancies of SLT use [17]. Self-reward while considered as an efficacious incentive to change behaviour [50], did not appear to be influential among our clients. Likewise, while there has been some success in challenging people’s perceptions by proposing that risky smoking behaviours are not accepted social norms [51]; this approach did not resonate with the clients using SLT. This might be because SLT use, unlike cigarette smoking, is still a social norm in certain sub-cultures to which our clients belonged [52]. Contrary to the standard practice, our advisors in the UK were reluctant to advice abrupt cessation, which they considered as a potential barrier to client engagement. Given that the latest Cochrane review comparing reduction versus abrupt cessation concluded that both approaches can be effective [53], a gradual reduction approach needs further examination for quitting SLT. Finally, our clients did not relate to using numeric scales to gauge their readiness to or confidence in quitting. We don’t fully understand the reasons for this but suspect that it might be linked to poor health literacy in Pakistan and in minority ethic communities in the UK [54].

Our study has several strengths. This is one of the few studies attempting cultural adaptation of an intervention to address a behaviour that is steeped in respective socio-cultural context. As we modelled components of BCI, we also developed a fidelity index that mapped across the intervention theory. We measured fidelity and provided an analytical approach, which can be used not just to measure fidelity but also to assess the relative effectiveness of the different components of the intervention. By conducting implementation research, both in Pakistan and the UK, we enhanced the external validity of our study. It also provided opportunities to share experiences and study differences between the two populations. Finally, by tackling a health issue that primarily affects women of a minority ethnic group in the UK, the study addressed health inequalities and offered opportunities to engage with marginalised communities. Our study also had some limitations. We were limited in our theoretical adaptation of BCTs for South Asian clients, by a handful of studies. Furthermore, we did not collect primary data on community perceptions directly from our target population before developing the intervention and rather relied on the literature reviews. Our sample size was small to make any statistical inference, albeit sufficient for an in-depth feasibility study of this nature. Our method of observation was based on audio-recordings only and could have missed visual clues on rapport building and body language between clients and the advisors. We were mindful of but could not account for, the differences between contrasting health-systems in Pakistan and in the UK. While the UK study sites were offering an on-going tobacco cessation service for SLT users, no such service existed in Pakistan. Likewise, NRT, although not part of BCI, was available to the clients in the UK. This was not the case in Pakistan. Due to the absence of a cotinine-testing lab in Pakistan, we were limited to using cotinine strips to assess SLT use there. While we understood that there are dual tobacco users (more among men than women of South Asian-origin), we did not include them in our study. However, we propose that integrating BISCA intervention with smoking cessation intervention and testing it in dual users would be an important and logical next step.

## Conclusions

 We expect to have developed a culturally appropriate behavioural intervention that could prove to be an effective tool in helping SLT users of South Asian-origin in quitting SLT use. Our study provides a methodological basis for designing future randomised controlled trials evaluating a behaviour change intervention for SLT cessation either alone or in combination with other pharmacological agents. Ours’ was part of the very first cohort of studies funded by the UK Medical Research Council’s new Public Health Intervention Development Scheme aimed at stimulating innovation in public health. We hope to have made a positive contribution to the overall aims of this programme by advancing methods for culturally adapting complex interventions, and assessing their fidelity, acceptability, and appropriateness.

## Abbreviations

BCI, Behaviour Change Intervention; BCT, Behaviour Change Technique; DALYs, Disability Adjusted Life Years; FTND-ST, Fagerstörm Tobacco and Nicotine Dependency scale for Smokeless Tobacco; NICE, National Institute of Health and Care Excellence; NRT, Nicotine Replacement Therapy; OSSTD, Oklahoma Scale for Smokeless Tobacco Dependence (OSSTD); SD, Standard Deviation; SLT, Smokeless Tobacco

## Additional files

Additional file 1: Topic guides to carry out interviews with service providers and patients. (PDF 144 kb) Additional file 2: Data on fidelity to Behavioural Change Intervention. (PDF 79 kb)
