# Supplementary material for: Behaviour change intervention for smokeless tobacco cessation: its development, feasibility and fidelity testing in Pakistan and in the UK
Source: BMC Public Health. 2016 Jun 10;16:501. doi: 10.1186/s12889-016-3177-8 (PMC4902895; doi:10.1186/s12889-016-3177-8)
Supplement: Additional file 1: — Topic guides to carry out interviews with service providers and patients. (PDF 144 kb) [file 12889_2016_3177_MOESM1_ESM.pdf]

Cessation Advisors

Interview Topic Guide

Process

1. **Introductions (if needed):** Name of researcher, name of Advisor

2. **Information Sheet and Consent Form:** Re-confirm that the Advisor is willing to take part in an interview (consent has been previously collected). Provide the opportunity for the Advisor to ask any further questions about the interview. Re-confirm consent to record the interview (if not take notes).

3. **Interview:** Ask questions below.

4. **Thank Advisor**

1. Background information (also act as ice breaker questions)

Can you tell me how long you have been working in smokeless tobacco cessation?

How are new clients bought forward for your service?
Prompt

- Are they referred or do you do outreach work?
- (If referred) Who makes the referrals (self/ HCP)?
- (If outreach) In what types of places do you promote the service?

In the absence of standardised guidelines, how have you previously supported clients with quitting smokeless tobacco?
Prompt

- Are there any specific strategies that you have previously used that have been particularly successful in supporting clients to quit? What are they?
- Any strategies that have not worked? What are they?
- (If they mention NHS assessment forms) Are these completed as part of your standard practice for smokeless tobacco users? How useful do you find this (why/why not)?

2. Delivering the resource

Now that you have used the new resource with several clients I'd like to hear how that has gone for you. There are no right or wrong answers I am simply interested in what you thought, so please do be honest.

How many clients have you used it with?

Overall (across the 3 sessions – pre-quit, quit and post-quit) what has been your experience of using the resource?
Prompt

- What has gone well?
- What has gone less well?

I'd like to talk about each session in turn (pre-quit, quit, post-quit).
Have each the advisor handbook and client cards available to discuss.

(a) PRE-QUIT SESSION

(Talk through each section below using advisor handbook and relevant cards)

How long did the pre-quit sessions take?
Prompt

- Did you have enough time? Why is that?
- How long would you ideally need?

Talking about smokeless tobacco products

The first thing you did with each client was to talk about smokeless tobacco products that they use and the risks of using them.

How did you find discussing smokeless tobacco products with your clients?
(Go through activities 1-4 in advisor handbook)
Prompt

- What worked well?
- What worked less well?
- How could we improve these activities?

You may have used *Red cards 1-4.*
Is there anything about any of these cards that you think we should change?
Prompt

- What worked well with them/less well?
- How should we change them – pictures/words?
- What else could we put in the pictures?
- What is your view on using pictures of people with oral cancer (or similar frightening images)?

Talking about stopping using ***smokeless tobacco***

Next you talked about how important it is for the client to stop using smokeless tobacco.

How did you find discussing these issues with your clients?
(Go through activities 5-8 in advisor handbook)
Prompt

- What worked well?
- What worked less well?
- How could we improve these activities?

You may have used *Red cards 5-7.*
Is there anything about any of these cards that you think we should change?
Prompt

- What worked well with them/less well?
- How should we change them – pictures/words?
- What else could we put in the pictures?
- What did you think about the pictures of money and the toothbrush?

Making plans to stop using smokeless tobacco

Finally you talked about making plans to stop using smokeless tobacco.

How did you find discussing these issues with your clients?
(Go through activities 9-15 in advisor handbook)
Prompt

- What worked well?
- What worked less well?
- How could we improve these activities?

You may have used *Red cards 8-11.*
Is there anything about any of these cards that you think we should change?
Prompt

- What worked well with them/less well?
- How should we change them – pictures/words?
- What else could we put in the pictures?

Nicotine Replacement Therapy

Did you talk about NRT within the pre-quit session?
Prompt

- How did you include it? (i.e. when in the session)
- Why did you/did you not talk about NRT?

Reflecting overall on Pre-quit sessions

Thinking overall about the pre-quit sessions you have done. How easy or difficult was the session to deliver? Why?
Prompt
- How would you change the session? (content, order delivered, resources etc.)
- Is there anything missing from the session? What?

Is here anything else about the pre-quit session that you want to tell me that you think could improve it?

(b) QUIT SESSION(S)

QUESTIONS FOR ADVISORS WHO HAVE DELIVERED THE QUIT SESSION AS PER PROTOCOL

(Talk through each section below using advisor handbook and relevant cards)

How long did the quit sessions take?
Prompt
- Did you have enough time? Why is that?
- How long would you ideally need?

Readiness to quit

The first thing you did with each client was to talk about the client's readiness to quit smokeless tobacco products

How did you find discussing this with your clients?
(Go through activity 1 in advisor handbook)
Prompt
- What worked well?
- What worked less well?
- How could we improve these activities?

You may have used *Green card 1*
Is there anything about this card that you think we should change?
Prompt
- What worked well with them/less well?
- How should we change them – pictures/words?
- What else could we put in the pictures?

New image as ex-user

You may then have discussed with clients the benefits of now being an ex-user.
How did you find discussing this with your clients?
(Go through activity 2 in advisor handbook)
Prompt
- What worked well?

- What worked less well?
- How could we improve this activity?

You may have used <u>*Green card 2*</u>
Is there anything in this card that you think we should change?
Prompt

- What worked well/less well?
- How – pictures/words?
- What else could we put in the pictures?

Withdrawal symptoms

How did you find discussing craving symptoms/triggers with your clients?
<u>*(Go through activities 3 and 4 in advisor handbook)*</u>
Prompt

- What worked well?
- What worked less well?
- How could we improve these activities?

Monitoring progress

Finally you may have discussed with clients the calendar for self-monitoring progress.
How did you find discussing this with your clients?
<u>*(Go through activity 5 in advisor handbook)*</u>
Prompt

- What worked well?
- What worked less well?
- How could we improve this activity?

Is there anything about calendar that you think we should change?
Prompt

- What worked well/less well?
- How – pictures/words?

Nicotine Replacement Therapy

Did you talk about NRT within the quit session?
Prompt
How did you include it? (i.e. when in the session)
Why did you/did you not talk about NRT?

Reflecting overall on Quit sessions
Thinking overall about the quit sessions you have done. How easy or difficult was the session to deliver? Why?
Prompt

- How, would you change the session? (content, order delivered, resources etc.)
- Is there anything missing from the session? What?

Is here anything else about the quit session that you want to tell me that you think could improve it?

QUESTIONS FOR ADVISORS WHO HAVE DELIVERTED ALTERNATIVE TYPES OF QUIT SESSIONS

Can you tell me how you have worked with clients after their first (pre-quit) session up to the time that they quit?

Prompt

- How many times do you typically see them?
- How often? (How is that decided?)

What do you cover in those sessions? *(discuss each session in turn where possible)*

Prompt

- What do you talk about? (reducing use gradually / readiness to quit / new image / withdrawal symptoms/ triggers / monitoring progress / other?)
- How do you decide what you cover with each client?

How do you use nicotine replacement therapies in these sessions?

Prompt

- How do you decide if/how to use NRT with each client?

Thinking about these sessions what works well / less well?

Prompt

- How might you improve these sessions?

(c) POST QUIT SESSION

QUESTIONS FOR ADVISORS WHO HAVE DELIVERED THE POST QUIT SESSION AS PER PROTOCOL

(Talk through each section below using advisor handbook and relevant cards)

How long did the post-quit sessions take?
Prompt
- Did you have enough time? Why is that?
- How long would you ideally need?

Abstinence Status
The first thing you did with each client was to talk about the client's abstinence status.

How did you find discussing this with your clients?
(Go through activity 1 in advisor handbook)
Prompt
- What worked well?
- What worked less well?
- How could we improve this activity?

How helpful/or not was the calendar in doing this?
Prompt
Had clients completed it correctly? If not, why not? What should we change?

Provide reward (if client stayed quit for 4 weeks)
If the client had quit for 4 weeks you discussed the reward that had been agreed in the quit session.

How did you find discussing this with your clients?
(Go through activity 2 in advisor handbook)
Prompt
- What worked well?
- What worked less well?
- How could we improve this activity?

Craving symptoms
You then discussed any difficult situations the client may have experienced.

How did you find discussing this with your clients?
(Go through activity 3 in advisor handbook)
Prompt
- What worked well?
- What worked less well?
- How could we improve this activity?

Nicotine Replacement Therapy

Did you talk about NRT within the post-quit session?
Prompt
How did you include it? (i.e. when in the session)
Why did you/did you not talk about NRT?

Reflecting overall on Post-quit sessions

Thinking overall about the post-quit sessions you have done. How easy or difficult was the session to deliver? Why?
Prompt

- How, would you change the session? (content, order delivered, resources etc.)
- Is there anything missing from the session? What?

Is here anything else about the pre-quit session that you want to tell me that you think could improve it?

Nicotine Replacement Therapy

Did you talk about NRT within any of the sessions?
Prompt

- How did you include it? (i.e. when in the session)
- Why did you/did you not talk about NRT?

3. Impact and Sustainability

How much prior knowledge on smokeless tobacco use did you have?
Has delivering this resource changed your knowledge? How?

How much prior knowledge on behaviour change techniques did you have?
Has delivering this resource changed your knowledge? How?

How confident (scale of 1 -10) are you now in supporting smokeless tobacco clients to quit?
Prompt

- Why is that?

How likely (scale of 1-10) are you to continue to use this resource with smokeless tobacco users?
Prompt

- Why is that? (will they change it/keep the same?)
- What will help you to include this resource within your routine service?
- What will make it difficult to include it within your routine service?

4. Training needs

Having now used this resource with several clients, how useful was the training you attended in York?
Prompt

- How helpful was it in supporting you to deliver the resource? Which elements?
- Is there any other training that would have been useful?
- How can we improve the training?

5. Final question

Finally, is there anything else you want to tell us about your experience of delivering the smokeless tobacco cessation resource?

QUESTIONS FOR ADVISORS WHO HAVE DELIVERED ALTERNATIVE TYPES OF POST-QUIT SESSIONS

Can you tell me how you have worked with clients after they have quit?
Prompt

- How many times do you typically see them?
- How often? (How is that decided?)

What do you cover in those sessions? *(discuss each session in turn where possible)*
Prompt

- What do you talk about? (abstinence status, rewards, craving symptoms))
- How do you decide what you cover with each client?

(How) do you use nicotine replacement therapies in these sessions?
Prompt

- How do you decide if/how to use NRT with each client?

Thinking about these sessions what works well / less well?
Prompt

- How might you improve these sessions?
