# Supplementary material for: Behaviour change intervention for smokeless tobacco cessation: its development, feasibility and fidelity testing in Pakistan and in the UK
Source: BMC Public Health. 2016 Jun 10;16:501. doi: 10.1186/s12889-016-3177-8 (PMC4902895; doi:10.1186/s12889-016-3177-8)
Supplement: Additional file 2: — Data on fidelity to Behavioural Change Intervention. (PDF 79 kb) [file 12889_2016_3177_MOESM2_ESM.pdf]

Additional file 4: Data on fidelity to Behavioural Support Intervention

**Behavioural Support Intervention ingredients**	**Constituent BCTs**	**Implementation status N (%) receiving the ingredient** (Sample N= 32)			
		Fully implemented	Partially implemented	Not implemented	Missing information
**Adherence to content**					
Identify product	RI1: Assess current and past tobacco use behaviour	27 (84.4)	5 (15.6)	0	0
Explain what the product contains	BM1: Provide information on consequences of tobacco use	24 (75.0)	7 (21.9)	1 (3.1)	0
Identify harmful ingredients	BM1: Provide information on consequences of tobacco use<br>5.2: Salience of consequences	24 (75.0)	5 (15.6)	3 (9.4)	0
Myths	5.1: Information about health consequences of tobacco use<br>5.2: Salience of consequences<br>4.3: Re-attribution: elicit perceived causes of behaviour and suggest alternative explanations	27 (84.4)	5 (15.6)	0	0
Importance scale	RI2: Assess current readiness and ability to stop<br>BM9: Identify reasons for wanting and not wanting to stop tobacco use	19 (59.4)	12 (37.5)	1 (3.1)	0
Benefits of stopping	BM9: Identify reasons for wanting and not wanting to stop tobacco use	24 (75.0)	8 (25.0)	0	0

	13.2: Framing/re-framing: Suggest the deliberate adoption of a perspective on behaviour (e.g. its purpose) in order to change cognitions or emotions about performing the behaviour				
Assess past quit attempts	RI3: Assess past history of quit attempts<br>BM2: Boost motivation and self-efficacy<br>15.3: Focus on past success	20 (62.5)	3 (9.4)	8 (25.0)	1 (3.1)
Confidence scale	RI2: Assess current readiness and ability to stop<br>15.3: Focus on past success	19 (59.4)	12 (37.5)	1 (3.1)	0
Help is available	BM2: Boost motivation and self-efficacy<br>BM10: Explain the importance of abrupt cessation<br>RC4: Explain expectations regarding treatment programme<br>15.3: Focus on past success	19 (59.4)	6 (18.8)	6 (18.8)	1 (3.1)
Social norms	BM5: Provide normative information about others behaviour and experiences<br>4.3: Re-attribution	16 (50.0)	6 (18.8)	10 (31.3)	0
Set quit date (preparation and planning)	BM6: Prompt commitment from the client there and then<br>BS3: Facilitate action planning/develop treatment plan<br>BS8: Advise on environmental restructuring<br>12.3: Avoidance/reducing exposure to cues for the behaviour<br>4.4: Instruction on how to perform behaviour	20 (62.5)	5 (15.6)	7 (21.9)	0

Nicotine dependence and triggers information	Inform about the nature of nicotine dependence (no label)<br>Identify pattern of tobacco use (no label)<br>BS4: Facilitate goal setting<br>BS1: Facilitate barrier identification<br>4.2: Inform about antecedents	10 (31.3)	12 (37.5)	10 (31.3)	0
Managing triggers	BS3: Facilitate action planning/develop treatment plan<br>BS1: Facilitate problem solving<br>BS7: Advise on changing routine<br>8.2: Behaviour substitution<br>12.4: Distraction: Advise to use alternative focus for attention to avoid triggers for chewing<br>BS9: Advise on setting graded task	27 (84.4)	4 (12.5)	1 (3.1)	0
Setting rewards and offering client booklet	10.7: Self-incentive<br>RC5: Offer/direct towards appropriate written materials	16 (50.0)	13 (40.6)	3 (9.4)	0
Readiness scale	RI2: Assess current readiness and ability to stop<br>13.2: Framing and re-framing: Suggest that they might think of quitting as reducing the risk of consequences from continuing chewing (rather than just the benefits of stopping)	18 (56.3)	2 (6.3)	0	12 (37.5)
Strengthen ex-user identity	BM8: Strengthen ex-user identity<br>13.2: framing/re-framing: Encourage them to reframe in their mind why they wanted to stop in	18 (56.3)	0	2 (6.3)	12 (37.5)

	the first place<br>13.1: Encourage identification of self as a role model 'who used to chew': Inform that their own behaviour may be an example to others				
Withdrawal symptoms information	RC6: Provide information on withdrawal symptoms	15 (46.9)	5 (15.6)	0	12 (37.5)
Managing withdrawals	BS1: Facilitate barrier identification and problem solving<br>BS3: Facilitate action planning/develop treatment plan<br>BS4: Facilitate goal setting<br>8.2: Behaviour substitution<br>12.4: Distraction<br>15.4: Self-talk (positive)	18 (56.3)	0	2 (6.3)	12 (37.5)
Monitoring progress	BS4: Facilitate goal setting<br>BS6: Prompt self-recording	19 (59.4)	0	1 (3.1)	12 (37.5)
Determine clients abstinence status	BM2: Boost motivation and self-efficacy	18 (56.3)	1 (3.1)	1 (3.1)	12 (37.5)
Provide rewards	10.9: Self-reward	17 (53.1)	0	3 (9.4)	12 (37.5)
Discuss withdrawal symptoms	RI4: Assess withdrawal symptoms<br>BS2: Facilitate relapse prevention and coping	15 (46.9)	3 (9.4)	2 (6.3)	12 (37.5)
**Quality of delivery**					
Establish a positive , friendly and professional relationship with the client and foster a sense that the client's	RC1: Build general Rapport	25 (78.1)	5 (15.6)	2 (6.3)	0

experiences are understood					
Give general reassurance to the client that his/her experiences are normal and time limited	RC10: Provide reassurance	24 (75.0)	0	8 (25.0)	0
Prompt questions from the client and answer clearly and accurately	RC2: Elicit and answer Questions	24 (75.0)	6 (18.8)	2 (6.3)	0
Adopt a style of interaction that involves listening carefully to the client and where appropriate reflecting back to the client key elements of what s/he is saying	RC7: Use reflective listening	21 (65.6)	3 (9.4)	8 (25.0)	0
Provide summary of information exchanged and establish a clear confirmation of decisions made and commitments entered into	RC9: Summarise info and confirm client decisions	22 (68.8)	1 (3.1)	9 (28.1)	0
Use relevant information from the client to tailor the behavioural support provided/ flexible adaptation that takes into account individual patient needs	RD1: Tailor interactions appropriately	22 (68.8)	2 (6.3)	8 (25.0)	0
Emphasise client choice within the bounds of evidence based practice	RD2: Emphasise client choice	21 (65.6)	1 (3.1)	10 (31.3)	0
